# A new method for analyzing non-uniform guiding structures

**DOI:** 10.1038/s41598-023-30145-6

**Published:** 2023-02-17

**Authors:** Negar Yasi, Mahdi Boozari, Mohammad G. H. Alijani, Mohammad H. Neshati

**Affiliations:** 1 Electrical Department, Amirkabir University of Technology, Tehran, Iran; 2 Electrical Department, Ferdowsi University of Mashhad, Mashhad, Iran

**Keywords:** Engineering, Electrical and electronic engineering

## Abstract

This paper presents a new technique for analyzing a non-uniform transmission line (NUTL). This method expands the differential equations of voltage and current as the slope functions. Then, differential equations are solved using the explicit Runge–Kutta technique with the fourth-order method with four stages. It is shown that the proposed method can be used not only for analyzing the NUTL but also for analyzing the no-uniform waveguides (NUWG). Additionally, it is shown that the sensitivity of the proposed method concerning the discretization error is suitable. Several theoretical and practical NUTLs and NUWG are investigated to verify the proposed method’s accuracy and advantages. The performance of the proposed method is compared with those obtained by the other popular techniques, such as uniform cascaded sections (UCS).

## Introduction

The non-uniform Transmission lines are widely used in numerous applications such as electrical resonators^1^, hybrid amplifiers^2^, frequency synthesizers^3^, pulse shaping circuits^4^, antenna array beamforming networks^5^, and coupled microstrip lines^6^. Transmission lines made of the non-uniform profile are classified into two groups. The first category in which the applied lines are not parallel consists of power transmission lines, which are crossed over the valleys or are parts of complicated structures such as metallic towers. The second category consists of parallel lines, but generally, the profile of the conductors is not uniform. These include planar impedance matching networks with exponential profiles and stepped-impedance microwave filters and etc.^7–9^.

A few analytical methods are presented for the study of non-uniform TLs made by a particular profile, such as the TLs with the exponential^10^, linearly tapered^11^, and power-law^12^ profiles. One classical method for analyzing a NUTL is segmenting the structure into a cascade of uniform sections^13,14^. In^15^, an iterative perturbation technique is introduced for the analysis of NUTLs. Furthermore, a few numerical techniques are developed for analyzing the non-uniform TLs. However, most of them suffer from high computational costs and a slow convergence rate^16^.

Due to the mentioned importance of non-uniform structures in several microwave, antenna, and electromagnetic compatibility applications, their investigation still remains an active field of research in electrical engineering. Hence, in this paper, the explicit Runge–Kutta based technique is introduced to analyze non-uniform TLs and waveguides.

In this method, using the discretization technique and expanding the slope functions from differential equations describing voltage and current waves, a system of equations is derived. By solving the obtained system of equations, the magnitude and phase of the voltage and current propagating along the structure are computed. The accuracy of this method is dependent on the per-unit-length impedance and admittance. It is demonstrated that knowing the profile function allows one to easily calculate the per-unit-length parameters for most practical structures. The proposed method's performance is verified by examining several practical test cases. Also, it is shown that the computational cost of the introduced method is low. The proposed method can be only applied to a single non-uniform structure. So, this can be considered as a drawback of it. Probably, it can be developed for a coupled NUTL. However, this work in the present form is focused on a single NUTL.

## Theoretical formulation

**Figure 1:**
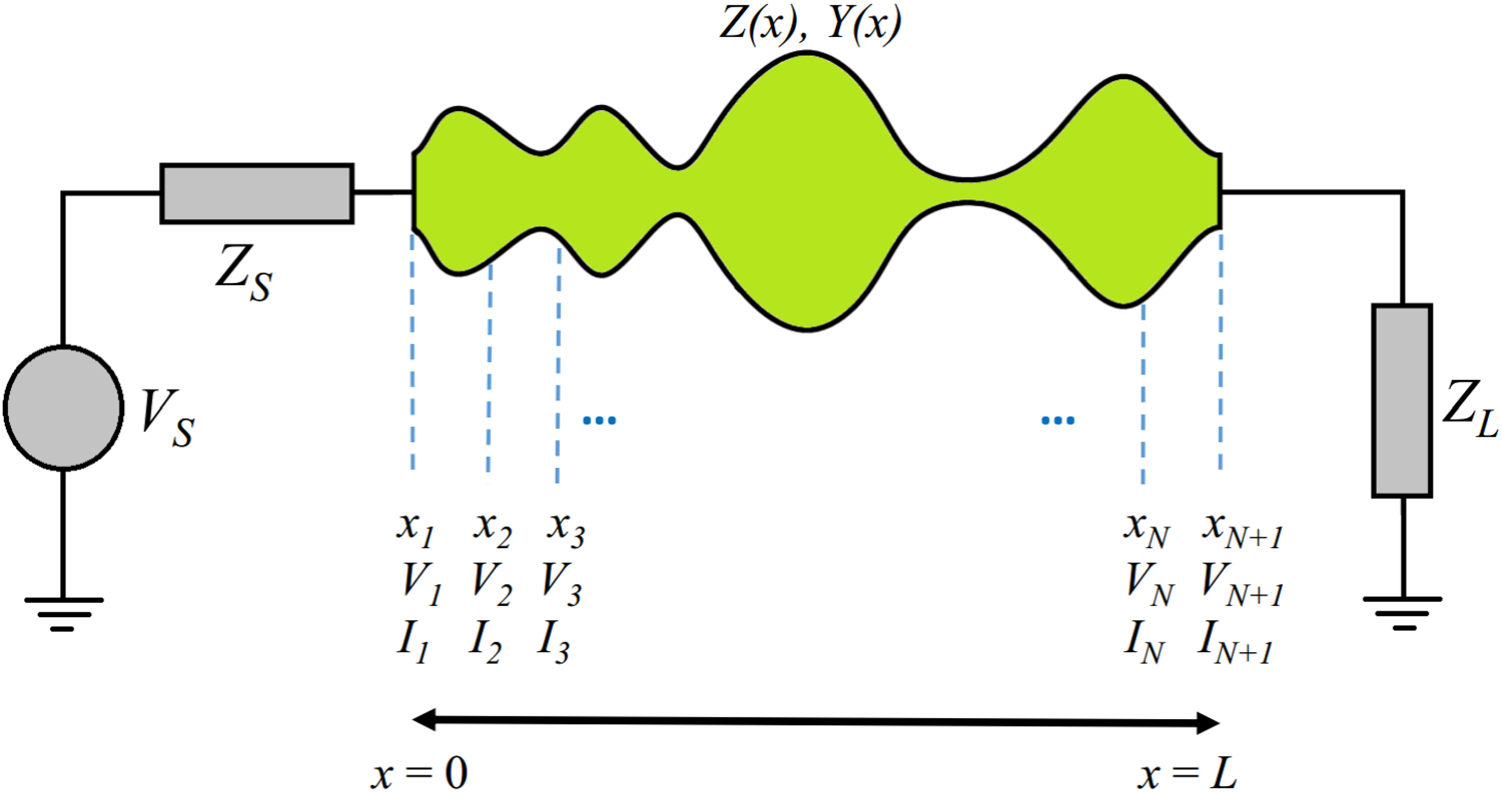
A non-uniform TL with general profile.

Figure 1 shows a non-uniform TL, which conveys TEM or quasi-TEM modes. It should be noted that the term “non-uniform” refers to a guiding structure with any arbitrary shape along the propagation direction. According to Fig. 1, the under-studying structure with length *L* is divided into *N* sections (*N* + 1 points). The voltage and current of any arbitrary point *x*_*n*_ (*n* = 1, …, *N* + 1) are depicted by *V*_*n*_ and *I*_*n*_, respectively.

Inspired by the explicit Runge–Kutta (RK) method^18^, voltage and current waves of the line are expressed by Eqs. (1a) and (1b), in which voltage and current at the point *x*_*n*_ are depicted by *V*_*n*_ and *I*_*n*_, respectively. As shown in Fig. 1, the voltage and current of each point is expressed by the following equations.

1a $$V_{n + 1} = V_{n} + h\sum\nolimits_{s = 1}^{S} {b_{s} k_{s} } , \, n = 1, \, 2, \, ..., \, N$$

1b $$I_{n + 1} = I_{n} + h\sum\nolimits_{s = 1}^{S} {a_{s} l_{s} } , \, n = 1, \, 2, \, ..., \, N$$

The parameters *h*, *S*, *b*_*s*_, *a*_*s*_, *k*_*s*_ and *l*_*s*_ are non-negative real constants called the step length of the method, the number of stages, weight of the voltage, weight of the current, the slope coefficients of the voltage, and the slope coefficients of the current, respectively. The above equations are a discrete expansion of the coupled version of the voltage and current differential equations of a non-uniform TL as follows^17^.

2a $${{dV} \mathord{\left/ {\vphantom {{dV} {dx}}} \right. \kern-0pt} {dx}} = - Z(x)I(x)$$

2b $${{dI} \mathord{\left/ {\vphantom {{dI} {dx}}} \right. \kern-0pt} {dx}} = - Y(x)V(x)$$

In the above equations, *V*(*x*) and *I*(*x*) are voltage and current waves, which propagate along the line with the length of *L*. *Z*(*x*) and *Y*(*x*) are per unit length impedance and per unit length admittance of the lines. Since both *Z*(*x*) and *Y*(*x*) are functions of *x*, an analytical solution for these waves is not found easily. It should be noted that for a TL, the voltage and current is a function of position x in the phasor domain; because it is assumed that the waves are propagating along the x-axis. So, the propagating waves only depends on *x*.

Selecting a proper value of the number of stages *S* is important to obtain a solution with reasonable accuracy. Selecting the higher values of *S* increases the accuracy and computational cost, simultaneously. So, there is a trade-off between the accuracy of the final response and the computational cost. In^18^, it is shown that *S* = 4 is a suitable value for balancing the accuracy and computational cost. The mathematical proof of accuracy, stability, and convergence rate for *S* = 4 can be found in^18^. It should be noted that the mathematical proof of the mentioned items is long and out of the scope of this work. In this case, the local truncation error and the total accumulated error are on the order of *O*(*h*^5^) and *O*(*h*^4^), respectively. By this assumption, Eqs. (2a) and (2b) can be rewritten by Eqs. (3a) and (3b)^18^.

3a $$V_{n + 1} = V_{n} + {{\left( {k_{1n} + 2k_{2n} + 2k_{3n} + k_{4n} } \right)} \mathord{\left/ {\vphantom {{\left( {k_{1n} + 2k_{2n} + 2k_{3n} + k_{4n} } \right)} 6}} \right. \kern-0pt} 6}$$

3b $$I_{n + 1} = I_{n} + {{\left( {l_{1n} + 2l_{2n} + 2l_{3n} + l_{4n} } \right)} \mathord{\left/ {\vphantom {{\left( {l_{1n} + 2l_{2n} + 2l_{3n} + l_{4n} } \right)} 6}} \right. \kern-0pt} 6},$$

in which the following equations have to be satisfied.

4a $$\left\{ \begin{gathered} k_{1n} = - hZ(x_{n} )I_{n} \hfill \\ l_{1n} = - hY(x_{n} )V_{n} \hfill \\ \end{gathered} \right.$$

4b $$\left\{ \begin{gathered} k_{2n} = - hZ\left( {x_{n} + {h \mathord{\left/ {\vphantom {h 2}} \right. \kern-0pt} 2}} \right)\left( {I_{n} + {{l_{1n} } \mathord{\left/ {\vphantom {{l_{1n} } 2}} \right. \kern-0pt} 2}} \right) \hfill \\ l_{2n} = - hY\left( {x_{n} + {h \mathord{\left/ {\vphantom {h 2}} \right. \kern-0pt} 2}} \right)\left( {V_{n} + {{k_{1n} } \mathord{\left/ {\vphantom {{k_{1n} } 2}} \right. \kern-0pt} 2}} \right) \hfill \\ \end{gathered} \right.$$

4c $$\left\{ \begin{gathered} k_{3n} = - hZ\left( {x_{n} + {h \mathord{\left/ {\vphantom {h 2}} \right. \kern-0pt} 2}} \right)\left( {I_{n} + {{l_{2n} } \mathord{\left/ {\vphantom {{l_{2n} } 2}} \right. \kern-0pt} 2}} \right) \hfill \\ l_{3n} = - hY\left( {x_{n} + {h \mathord{\left/ {\vphantom {h 2}} \right. \kern-0pt} 2}} \right)\left( {V_{n} + {{k_{2n} } \mathord{\left/ {\vphantom {{k_{2n} } 2}} \right. \kern-0pt} 2}} \right) \hfill \\ \end{gathered} \right.$$

4d $$\left\{ \begin{gathered} k_{4n} = - hZ(x_{n} + h)(I_{n} + l_{3n} ) \hfill \\ l_{3n} = - hY(x_{n} + h)\left( {V_{n} + k_{3n} } \right) \hfill \\ \end{gathered} \right.$$

4e $$x_{n} = x_{1} + nh, \, n = 1,2, \ldots , \, N + 1$$

4f $$h = {L \mathord{\left/ {\vphantom {L N}} \right. \kern-0pt} N}.$$

Another critical parameter is the step length *h*. Obviously, by reducing *h,* the computational accuracy is increased. Moreover, reducing *h* may increase the round-off error^19^. There are three suggestions for step size, including the significant one* h* = λ/10, the middle step *h* = λ/20, and the minor step *h* = λ/40, in which λ is the wavelength corresponding to the highest frequency^19^. Although *h* = λ/40 produces the minimum truncation error compared to that of the other ones, the round-off error and, accordingly, the computational cost are increased. Our studies show that *h* = λ/20 is a proper choice for the most practical applications. So, the step length *h* is very smaller than the TL length *L*. In this case, the following approximation is acceptable.

5a $$Z(x_{n} + h) \simeq Z\left( {x_{n} + {h \mathord{\left/ {\vphantom {h 2}} \right. \kern-0pt} 2}} \right) \simeq Z_{n}$$

5b $$Y(x_{n} + h) \simeq Y\left( {x_{n} + {h \mathord{\left/ {\vphantom {h 2}} \right. \kern-0pt} 2}} \right) \simeq Y_{n}$$

So Eqs. (6a) and (6b) express voltage and current along the line.

6a $$V_{n + 1} = \alpha_{1n} V_{n} + \alpha_{2n} I_{n}$$

6b $$I_{n + 1} = \alpha_{3n} V_{n} + \alpha_{4n} I_{n}$$

in which *α*_*i*_’s are given by Eqs. (7a), (7b), (7c) and (7d).

7a $$\alpha_{1n} = 1 + {{\left( {k_{1vn} + 2k_{2vn} + 2k_{3vn} + k_{4vn} } \right)} \mathord{\left/ {\vphantom {{\left( {k_{1vn} + 2k_{2vn} + 2k_{3vn} + k_{4vn} } \right)} 6}} \right. \kern-0pt} 6}$$

7b $$\alpha_{2n} = {{\left( {k_{1in} + 2k_{2in} + 2k_{3in} + k_{4in} } \right)} \mathord{\left/ {\vphantom {{\left( {k_{1in} + 2k_{2in} + 2k_{3in} + k_{4in} } \right)} 6}} \right. \kern-0pt} 6}$$

7c $$\alpha_{3n} = {{\left( {l_{1vn} + 2l_{2vn} + 2l_{3vn} v + l_{4vn} } \right)} \mathord{\left/ {\vphantom {{\left( {l_{1vn} + 2l_{2vn} + 2l_{3vn} v + l_{4vn} } \right)} 6}} \right. \kern-0pt} 6}$$

7d $$\alpha_{4n} = 1 + {{\left( {l_{1in} + 2l_{2in} + 2l_{3in} v + l_{4in} } \right)} \mathord{\left/ {\vphantom {{\left( {l_{1in} + 2l_{2in} + 2l_{3in} v + l_{4in} } \right)} 6}} \right. \kern-0pt} 6}$$

In the above equations, the following relations have to be satisfied.

8a $$\left\{ \begin{gathered} k_{1vn} = 0 \hfill \\ k_{1in} = - hZ_{n} \hfill \\ \end{gathered} \right., \, \,\,\,\,\,\,\left\{ \begin{gathered} l_{1vn} = - hY_{n} \hfill \\ l_{1in} = 0 \hfill \\ \end{gathered} \right.$$

8b $$\left\{ \begin{gathered} k_{2vn} = {{h^{2} Z_{n} Y_{n} } \mathord{\left/ {\vphantom {{h^{2} Z_{n} Y_{n} } 2}} \right. \kern-0pt} 2} \hfill \\ k_{2in} = - hZ_{n} \hfill \\ \end{gathered} \right., \, \,\,\,\,\,\,\left\{ \begin{gathered} l_{2vn} = - hY_{n} \hfill \\ l_{2in} = {{h^{2} Z_{n} Y_{n} } \mathord{\left/ {\vphantom {{h^{2} Z_{n} Y_{n} } 2}} \right. \kern-0pt} 2} \hfill \\ \end{gathered} \right.$$

8c $$\left\{ \begin{gathered} k_{3vn} = {{h^{2} Z_{n} Y_{n} } \mathord{\left/ {\vphantom {{h^{2} Z_{n} Y_{n} } 2}} \right. \kern-0pt} 2} \hfill \\ k_{3in} = - hZ_{n} - {{h^{3} Z_{n}^{2} Y_{n} } \mathord{\left/ {\vphantom {{h^{3} Z_{n}^{2} Y_{n} } 4}} \right. \kern-0pt} 4} \hfill \\ \end{gathered} \right.$$

8d $$\left\{ \begin{gathered} l_{3vn} = - hY_{n} - {{h^{3} Z_{n} Y_{n}^{2} } \mathord{\left/ {\vphantom {{h^{3} Z_{n} Y_{n}^{2} } 4}} \right. \kern-0pt} 4} \hfill \\ l_{3in} = {{h^{2} Z_{n} Y_{n} } \mathord{\left/ {\vphantom {{h^{2} Z_{n} Y_{n} } 2}} \right. \kern-0pt} 2} \hfill \\ \end{gathered} \right.$$

8e $$\left\{ \begin{gathered} k_{4vn} = - h^{2} Z_{n} Y_{n} + {{h^{4} Z_{n}^{2} Y_{n}^{2} } \mathord{\left/ {\vphantom {{h^{4} Z_{n}^{2} Y_{n}^{2} } 4}} \right. \kern-0pt} 4} \hfill \\ k_{4in} = - hZ_{n} - {{h^{3} Z_{n}^{2} Y_{n} } \mathord{\left/ {\vphantom {{h^{3} Z_{n}^{2} Y_{n} } 2}} \right. \kern-0pt} 2} \hfill \\ \end{gathered} \right.$$

8f $$\left\{ \begin{gathered} l_{4vn} = - hY_{n} - {{h^{3} Z_{n} Y_{n}^{2} } \mathord{\left/ {\vphantom {{h^{3} Z_{n} Y_{n}^{2} } 2}} \right. \kern-0pt} 2} \hfill \\ l_{4in} = h^{2} Z_{n} Y_{n} + {{h^{4} Z_{n}^{2} Y_{n}^{2} } \mathord{\left/ {\vphantom {{h^{4} Z_{n}^{2} Y_{n}^{2} } 4}} \right. \kern-0pt} 4} \hfill \\ \end{gathered} \right.$$

According to Fig. 1 and Eqs. (1a), (1b) and (2a), (2b), two boundary conditions can be considered as follows.

9a $$V\left( 0 \right) + Z_{S} I\left( 0 \right) = V_{S}$$

9b $$V\left( L \right) - Z_{L} I\left( L \right) = 0$$

In above equations, *V*(0), *I*(0), *V*(*L*), and *I*(*L*) are the same as *V*_*1*_, *I*_*1*_, *V*_*N*+1_, *I*_*N*+1_ in the discrete form, respectively. Then, the following linear system of equations is established using boundary conditions Eqs. (9a) and (9b).

10a $${\mathbf{AX}} = {\mathbf{B}}$$

10b $${\mathbf{A}} = \left[ {\begin{array}{*{20}c} {{\mathbf{A}}_{{{\mathbf{11}}}} } & {{\mathbf{A}}_{{{\mathbf{12}}}} } \\ {{\mathbf{A}}_{{{\mathbf{21}}}} } & {{\mathbf{A}}_{{{\mathbf{22}}}} } \\ \end{array} } \right]$$

10c $${\mathbf{X}} = \left[ {\begin{array}{*{20}c} {\mathbf{V}} & {\mathbf{I}} \\ \end{array} } \right]^{T}$$

10d $${\mathbf{V}} = \left[ {\begin{array}{*{20}c} {V_{1} } & {V_{2} } & \cdots & {V_{N + 1} } \\ \end{array} } \right]^{T}$$

10e $${\mathbf{I}} = \left[ {\begin{array}{*{20}c} {I_{1} } & {I_{2} } & \cdots & {I_{N + 1} } \\ \end{array} } \right]^{T}$$

10f $${\mathbf{B}} = \left[ {\begin{array}{*{20}c} {V_{S} } & 0 & \cdots & 0 \\ \end{array} } \right]_{1 \times (2N + 2)}^{T}$$

The coefficient matrix **A** is constructed using four blocks given by Eqs. (11a), (11b), (11c) and (11d). It should be noted that these blocks are a function of *n*. So, the elements of them need to be updated for each *n*.

11a $${\mathbf{A}}_{{{\mathbf{11}}}} = \left[ {\begin{array}{*{20}c} 1 & 0 & 0 & 0 & \cdots & 0 \\ {\alpha_{11} } & { - 1} & 0 & 0 & \cdots & 0 \\ 0 & {\alpha_{12} } & { - 1} & 0 & \cdots & 0 \\ \vdots & \vdots & \vdots & \vdots & \cdots & \vdots \\ 0 & \cdots & 0 & {\alpha_{1n} } & { - 1} & 0 \\ 0 & \cdots & 0 & 0 & {\alpha_{1N} } & { - 1} \\ \end{array} } \right]_{{\left( {N + 1} \right) \times \left( {N + 1} \right)}}$$

11b $${\mathbf{A}}_{{{\mathbf{12}}}} = \left[ {\begin{array}{*{20}c} {Z_{S} } & 0 & 0 & 0 & \cdots & 0 \\ {\alpha_{21} } & 0 & 0 & 0 & \cdots & 0 \\ 0 & {\alpha_{22} } & 0 & 0 & \cdots & 0 \\ \vdots & \vdots & \vdots & \vdots & \cdots & \vdots \\ 0 & \cdots & 0 & {\alpha_{2n} } & 0 & 0 \\ 0 & \cdots & 0 & 0 & {\alpha_{2N} } & 0 \\ \end{array} } \right]_{{\left( {N + 1} \right) \times \left( {N + 1} \right)}}$$

11c $${\mathbf{A}}_{{{\mathbf{21}}}} = \left[ {\begin{array}{*{20}c} {\alpha_{31} } & 0 & 0 & 0 & \cdots & 0 \\ 0 & {\alpha_{32} } & 0 & 0 & \cdots & 0 \\ 0 & 0 & {\alpha_{3n} } & 0 & \cdots & 0 \\ \vdots & \vdots & \vdots & \vdots & \cdots & \vdots \\ 0 & \cdots & 0 & 0 & {\alpha_{3N} } & 0 \\ 0 & \cdots & 0 & 0 & 0 & 1 \\ \end{array} } \right]_{{\left( {N + 1} \right) \times \left( {N + 1} \right)}}$$

11d $${\mathbf{A}}_{{{\mathbf{22}}}} = \left[ {\begin{array}{*{20}c} {\alpha_{41} } & { - 1} & 0 & 0 & \cdots & 0 \\ 0 & {\alpha_{42} } & { - 1} & 0 & \cdots & 0 \\ 0 & 0 & {\alpha_{43} } & { - 1} & \cdots & 0 \\ \vdots & \vdots & \vdots & \vdots & \cdots & \vdots \\ 0 & \cdots & 0 & 0 & {\alpha_{4N} } & { - 1} \\ 0 & \cdots & 0 & 0 & 0 & { - Z_{L} } \\ \end{array} } \right]_{{\left( {N + 1} \right) \times \left( {N + 1} \right)}}$$

Finally, for an invertible matrix **A**, the unique solution of the system of equations is obtained by Eq. (12). It should be noted that there is no guarantee that this condition will be met. However, our studies show that for many practical applications, the coefficient matrix **A** is invertible.

12 $${\mathbf{X}} = {\mathbf{A}}^{ - 1} {\mathbf{B}}$$

As stated in^18^, the essential advantages of the Runge–Kutta method are its higher stability, mainly when it is applied to a practical system.

The conversion from voltages/currents to S-parameters is relatively standard and can be found in^17^. It is helpful to note that for symmetrical and reciprocal structures, *S*_*11*_ = *S*_*22*_, *S*_*12*_ = *S*_*21*_, and based on the conservation of power principle, |*S*_*11*_|^2^ +|*S*_*21*_|^2^ = 1^17^. According to Eqs. (10a), (10b), (10c), (10d), (10e) and (10f), the accuracy of the proposed method is also dependent on per-unit-length parameters. If there is no closed-form expression for *Z*(x) or *Y*(x) of the structure under study, a numerical technique has to be employed^13^. To this end, the per-unit-length parameters can be computed by the scalar potential *φ*(*x*) given by Eqs. (13) and (14)^20^.

13 $$Z(x) = \left( {{{j\omega \mu } \mathord{\left/ {\vphantom {{j\omega \mu } {2\pi }}} \right. \kern-0pt} {2\pi }}} \right)\varphi (x)$$

14 $$Y(x) = 2\pi \left[ {\sigma + j\omega \varepsilon } \right]\varphi^{ - 1} (x)$$

in which *μ*(*x*), *ε*(*x*), and *σ*(*x*) are permeability, permittivity and conductivity of the applied substrate, respectively.

## Extension to non-uniform waveguide

As shown in^21^, Eqs. (1a) and (1b) can also be used for a non-uniform waveguide, operating at its dominant mode. In this case, electric and magnetic fields along the waveguide can be equated by scalar voltage and current functions, which satisfy TL equations given by Eqs. (15a) and (15b).

15a $${{dI} \mathord{\left/ {\vphantom {{dI} {dx}}} \right. \kern-0pt} {dx}} = {{jkV} \mathord{\left/ {\vphantom {{jkV} \eta }} \right. \kern-0pt} \eta }$$

15b $${{dV} \mathord{\left/ {\vphantom {{dV} {dx}}} \right. \kern-0pt} {dx}} = {{j\eta \beta^{2} I} \mathord{\left/ {\vphantom {{j\eta \beta^{2} I} k}} \right. \kern-0pt} k}$$

in which

16a $$k = \omega \sqrt {\mu \varepsilon }$$

16b $$\eta = \sqrt {{\mu \mathord{\left/ {\vphantom {\mu \varepsilon }} \right. \kern-0pt} \varepsilon }} ,$$

*β* is phase constant of the propagation wave. For a non-uniform waveguide, *Z*(*x*) and *Y*(*x*) have to be known. For example, per-unit-length impedance and admittance of a rectangular waveguide with height *b* and non-uniform width *w*(*x*) at its dominant TE_10_ mode is found using Eqs. (17a) and (17c)^11^.

17a $$Z(x) = {{j\omega \mu b} \mathord{\left/ {\vphantom {{j\omega \mu b} {w(x)}}} \right. \kern-0pt} {w(x)}}$$

17b $$Y(x) = {{j\gamma^{2} (x)w(x)} \mathord{\left/ {\vphantom {{j\gamma^{2} (x)w(x)} {\omega \mu b}}} \right. \kern-0pt} {\omega \mu b}}$$

17c $$\gamma (x) = \sqrt {k^{2} - \left( {{\pi \mathord{\left/ {\vphantom {\pi {w(x)}}} \right. \kern-0pt} {w(x)}}} \right)^{2} } ,$$

in which *γ* is the propagating constant, and *k* is the free space wavenumber. For the other types of waveguides, similar formulas can be used.

## Results and discussion

In order to confirm the accuracy of the suggested method, some practical non-uniform TL structures are examined, and their results are compared to those obtained by measurement, simulation and UCS technique^13^. The simulation results are obtained by using High Frequency Structure Simulator (HFSS).

### Catenary transmission line

Catenary shaped-transmission lines are widely used in electrical engineering, especially in power systems. Determining voltage and current waves in a catenary TL is a challenging problem in these systems. The function *y*(*x*) describing the profile of a catenary TL is given by Eq. (18), in which *q* is the constant of the catenary line^22^.

18 $$y(x) = q\cosh \left( {{x \mathord{\left/ {\vphantom {x q}} \right. \kern-0pt} q} - {l \mathord{\left/ {\vphantom {l {2q}}} \right. \kern-0pt} {2q}}} \right)$$

The per-unit-length longitudinal impedance and the per-unit-length transversal admittance of a catenary TL are calculated by Eqs. (19a) and (19b)^22^.

19a $$Z(x) = \left( {{{j\omega \mu } \mathord{\left/ {\vphantom {{j\omega \mu } {2\pi }}} \right. \kern-0pt} {2\pi }}} \right)\cosh^{ - 1} \left( {{{2y(x)} \mathord{\left/ {\vphantom {{2y(x)} {r_{0} }}} \right. \kern-0pt} {r_{0} }}} \right)$$

19b $$Y(x) = \frac{j2\pi \omega \varepsilon }{{\cosh^{ - 1} \left( {{{2y(x)} \mathord{\left/ {\vphantom {{2y(x)} {r_{0} }}} \right. \kern-0pt} {r_{0} }}} \right)}}$$

**Figure 2:**
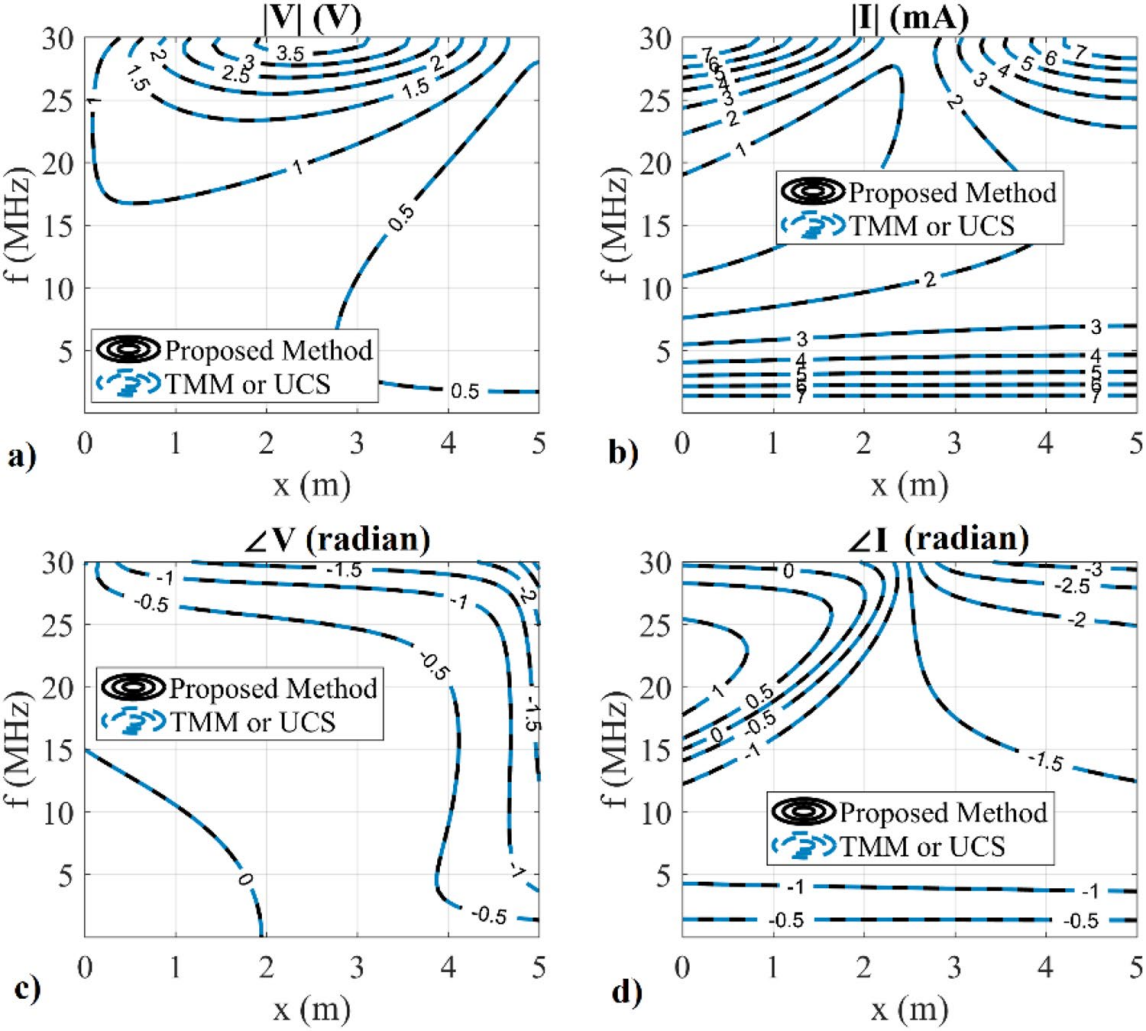
The calculated *V*(*x*) and *I*(*x*) of a catenary TL using the proposed and TMM, UCS techniques; (**a**) magnitude of *V*, (**b**) magnitude of *I*, (**c**) phase of *V*, (**d**) phase of *I*.

In the above equations, *l* and *r*_*0*_ are the line length and radius of the conductor, respectively. In the first example, a catenary TL with *q* = 3, *l* = 5 m, *r*_*0*_ = 5 mm, *V*_*S*_ = 1 V, *Z*_*L*_ = 75 Ω and *Z*_*S*_ = 50 Ω is considered. The analytical solution of the catenary TL is not available. However, the catenary TL can be analyzed using the Transfer Matrix Method (TMM)^23^ or UCS technique with excellent accuracy. The magnitude and phase of the calculated *V*(*x*) and *I*(*x*) using the proposed and TMM/UCS method are shown in Fig. 2 in contour format. In this figure, the variation of both magnitude and phase of *V*(*x*) and *I*(*x*) are plotted versus frequency and position simultaneously. A very excellent agreement can be seen between the results of the proposed and TMM/UCS methods over the operating range of 0 ≤ *x* ≤ 5 m and 0 ≤ *f* ≤ 30 MHz.

### Non-uniform microstrip filter

**Figure 3:**
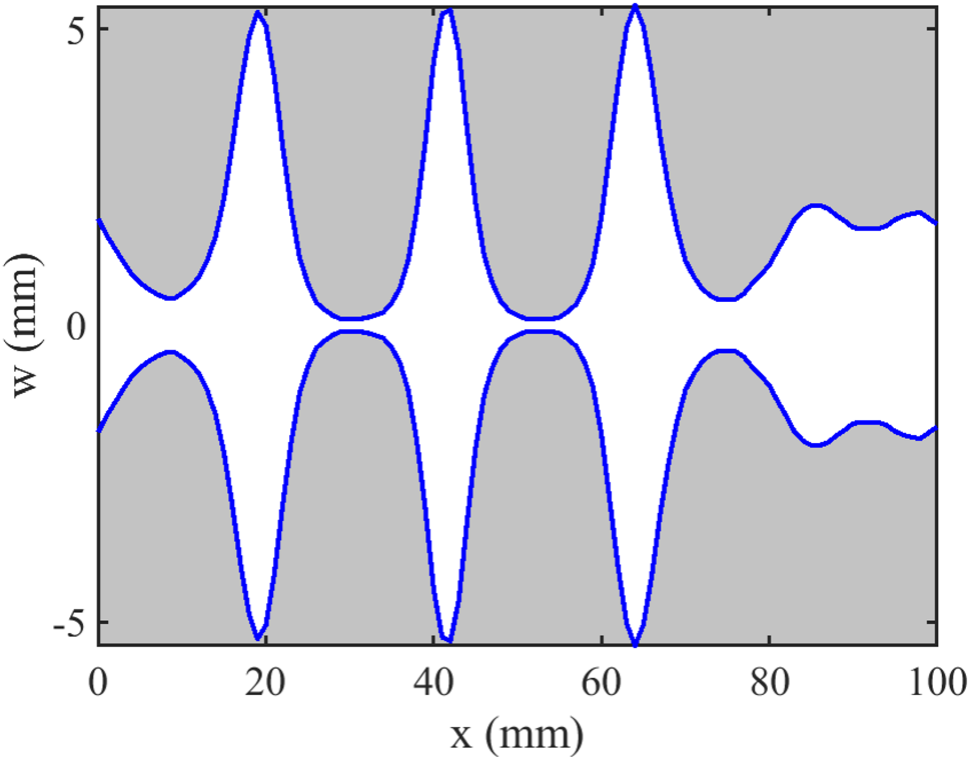
The profile of stop-band filter.

**Figure 4:**
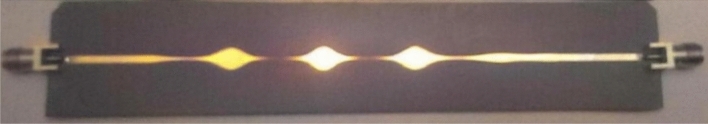
The picture of fabricated stop-band filter^24^.

Microstrip filters made with a non-uniform profile are widely used in microwave systems due to their low profile and low cost. A stop band filter based on a non-uniform microstrip line is proposed in^24^. The relative permittivity constant (*ε*_*r*_) and the thickness of the substrate (*H*) are 3.5 and 0.768 mm, respectively. The length of the non-uniform section is about 10 cm. The designed filter is connected to a 50 Ω impedance at two ports. The width (*W*) of the filter is varied as *x*. The function describing the profile of the filter is shown in Fig. 3. The picture of the fabrication filter is depicted in Fig. 4. At any arbitrary point *x*, the effective dielectric constant is calculated as^17^.

20 $$\varepsilon_{eff} \left( x \right) = \frac{{\varepsilon_{r} + 1}}{2} + \frac{{\varepsilon_{r} - 1}}{2}\frac{1}{{\sqrt {1 + 12H/W\left( x \right)} }}$$

The characteristic impedance can be calculated as a function of *x* using the introduced equations in^17^. After that, the per unit length capacitance *C*(*x*) and per unit length inductance *L*(*x*) are estimated as follows^17^.

21 $$\left\{ \begin{gathered} C\left( x \right) = {{\sqrt {\mu \varepsilon_{0} \varepsilon_{eff} \left( x \right)} } \mathord{\left/ {\vphantom {{\sqrt {\mu \varepsilon_{0} \varepsilon_{eff} \left( x \right)} } {Z_{C} \left( x \right)}}} \right. \kern-0pt} {Z_{C} \left( x \right)}} \hfill \\ L\left( x \right) = {{\mu \varepsilon_{0} \varepsilon_{eff} \left( x \right)} \mathord{\left/ {\vphantom {{\mu \varepsilon_{0} \varepsilon_{eff} \left( x \right)} {C\left( x \right)}}} \right. \kern-0pt} {C\left( x \right)}} \hfill \\ \end{gathered} \right.$$

**Figure 5:**
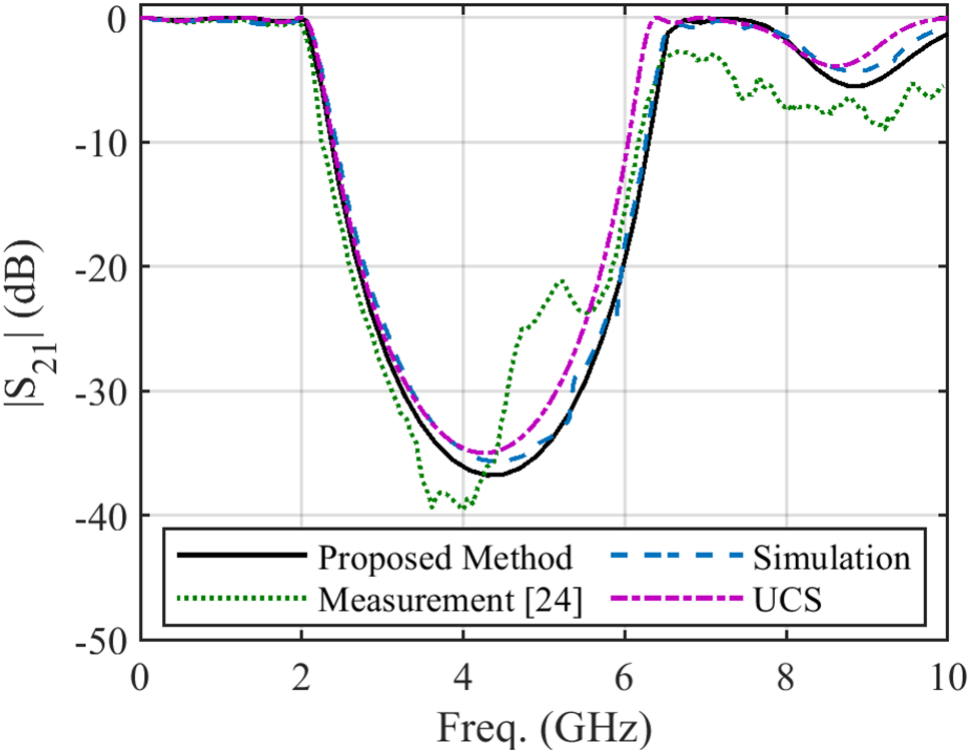
The frequency response of the non-uniform stop-band filter.

Finally, an approximation of the per unit length parameters can be determined as *Z*(*x*) = j*ωL*(*x*), *Y*(*x*) = j*ωC*(*x*). Figure 5 depicts the frequency response of the non-uniform filter. Over the frequency range 0 ≤ *f* ≤ 10 GHz, the accuracy of all results is approximately matched. At other frequencies, the results of the proposed method are similar to the simulation data. In general, the accuracy of the proposed method is acceptable over the wide frequency range of 0 ≤ *f* ≤ 10 GHz. It should be noted that the reflection coefficient is not reported in^24^.

### Non-uniform TL with sharp discontinuities

**Figure 6:**
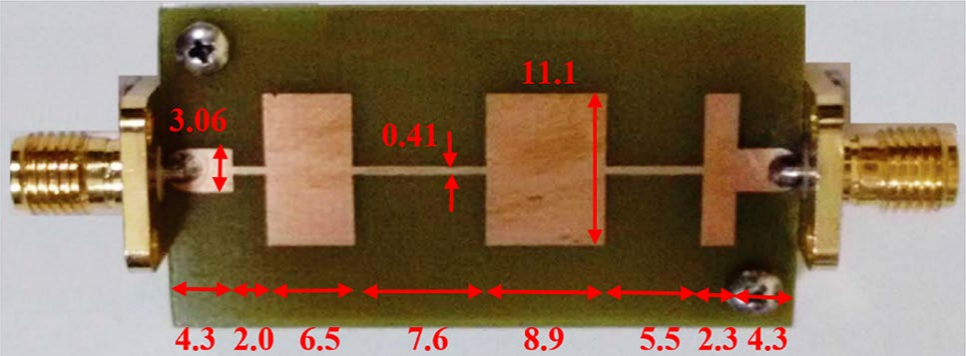
The picture of fabricated stepped-impedance filter (unit: mm)^25^.

**Figure 7:**
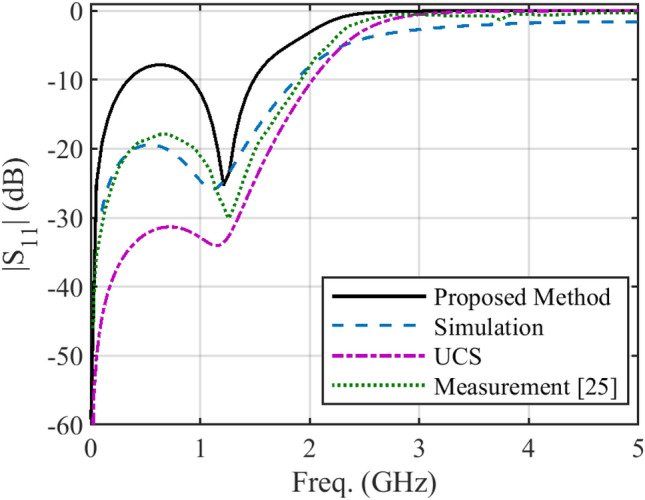
Magnitude of *S*_*11*_ of the stepped-impedance filter.

**Figure 8:**
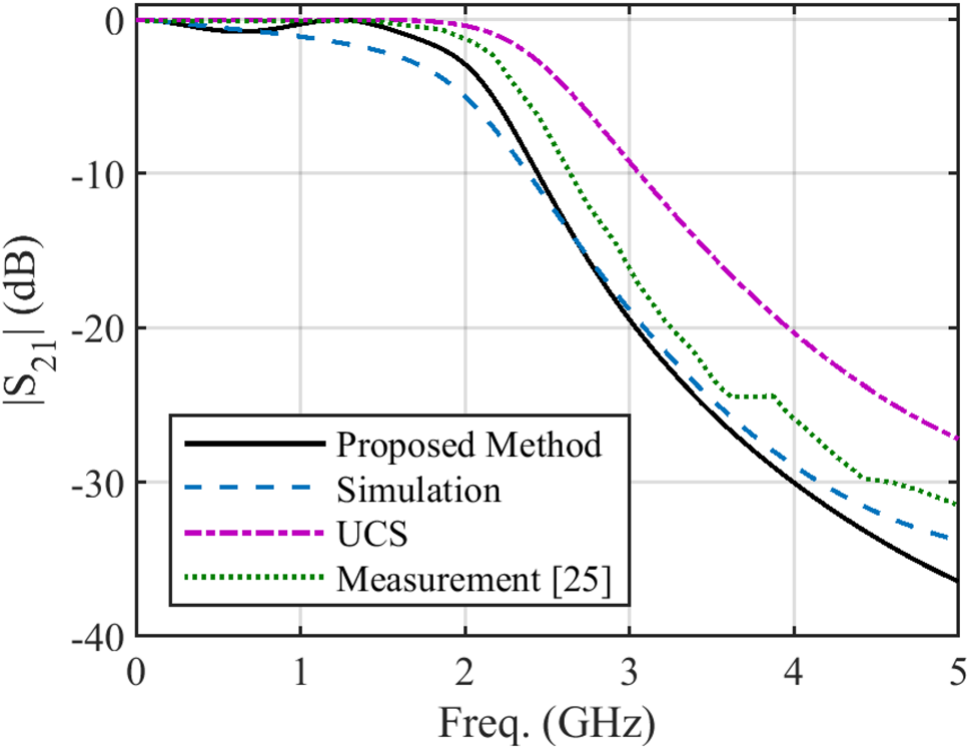
Magnitude of *S*_*21*_ of the stepped-impedance filter.

In some structures, there are several discontinuities in the profile of NUTL, such as the stepped-impedance microstrip filters. The third example considers a microstrip line with six sharp discontinuities in its profile. The relative permittivity constant, tangent loss and the thickness of the substrate are 4.4, 0.02, and 1.6 mm, respectively. This structure is terminated to 50 Ω impedances at two ports. The minimum and maximum width of the steps are 0.4080 mm and 11.1 mm, respectively. The per unit length parameters are determined using the introduced procedure for the previous example. Figure 6 shows the fabricated structure^25^. The scattering parameters of the under-studying structure are shown in Fig. 7 and 8. Similar to the previous example, these figures include the simulation and measurement data^25^ and the result of the UCS method. As seen in Fig. 7, the difference between the proposed and UCS results is high at the lower frequencies. It is probably due to the connector effect, which is not included in the calculating procedure. Also, for this structure, around the discontinuities locations, there would be higher-order modes, which are ignored in both proposed and UCS methods. In turn, at a higher frequency, the proposed and UCS methods’ accuracy is acceptable. The accuracy of the proposed method is superior to the UCS method at all frequencies, as shown in Fig. 8.

### Non-uniform substrate integrated waveguide

**Figure 9:**
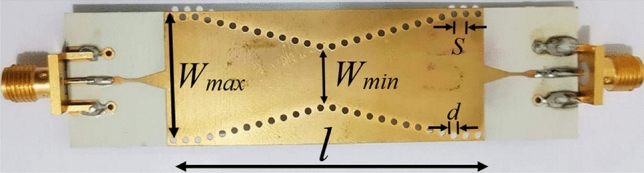
The picture of fabricated linearly-tapered SIW^11^.

A SIW structure with a non-uniform profile is investigated as the last example to verify the application of the proposed method in analyzing the NUWGs. The picture of fabricated double slope linearly-tapered SIW is displayed in Fig. 9^11^. The geometrical and physical parameters of this structure are *ε*_*r*_ = 3.66, tanδ = 0.0037, *h* = 0.254 mm, *l* = 44 mm, *Z*_*S*_ = *Z*_*L*_ = 50 Ω, *d* = 1 mm, *S* = 2 mm, *W*_*min*_ = 10 mm, and *W*_*max*_ = 20 mm. In a SIW structure with a width of *W*, the width of its equivalent rectangular waveguide is approximated by Eqs. (22a), (22b), (22c) and (22d)^11^.

22a $$W_{eff} = W\left( {a_{1} + \frac{{a_{2} }}{{{S \mathord{\left/ {\vphantom {S d}} \right. \kern-0pt} d} + \left( {{{\left( {a_{1} + a_{2} - a_{3} } \right)} \mathord{\left/ {\vphantom {{\left( {a_{1} + a_{2} - a_{3} } \right)} {\left( {a_{3} - a_{1} } \right)}}} \right. \kern-0pt} {\left( {a_{3} - a_{1} } \right)}}} \right)}}} \right)$$

22b $$a_{1} = 1.0198 + \frac{0.3465}{{{W \mathord{\left/ {\vphantom {W S}} \right. \kern-0pt} S} - 1.0684}}$$

22c $$a_{2} = - 0.1183 + \frac{1.2729}{{{W \mathord{\left/ {\vphantom {W S}} \right. \kern-0pt} S} - 1.2010}}$$

22d $$a_{3} = 1.0082 + \frac{0.9163}{{{W \mathord{\left/ {\vphantom {W S}} \right. \kern-0pt} S} + 0.2152}}$$

**Figure 10:**
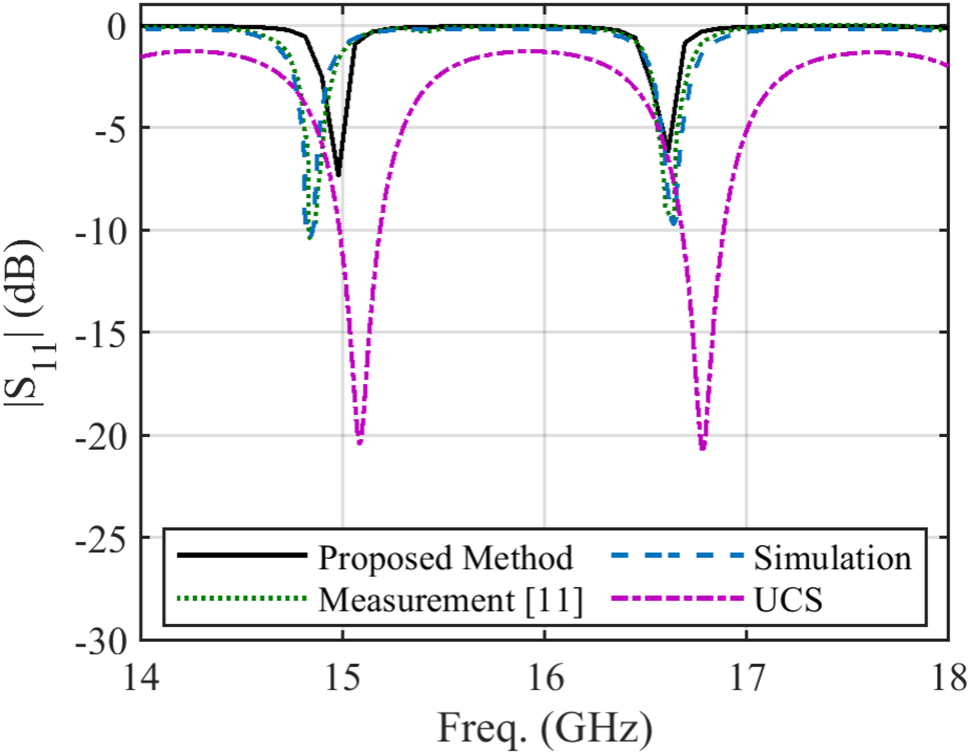
Magnitude of *S*_*11*_ of the linearly-tapered SIW.

**Figure 11:**
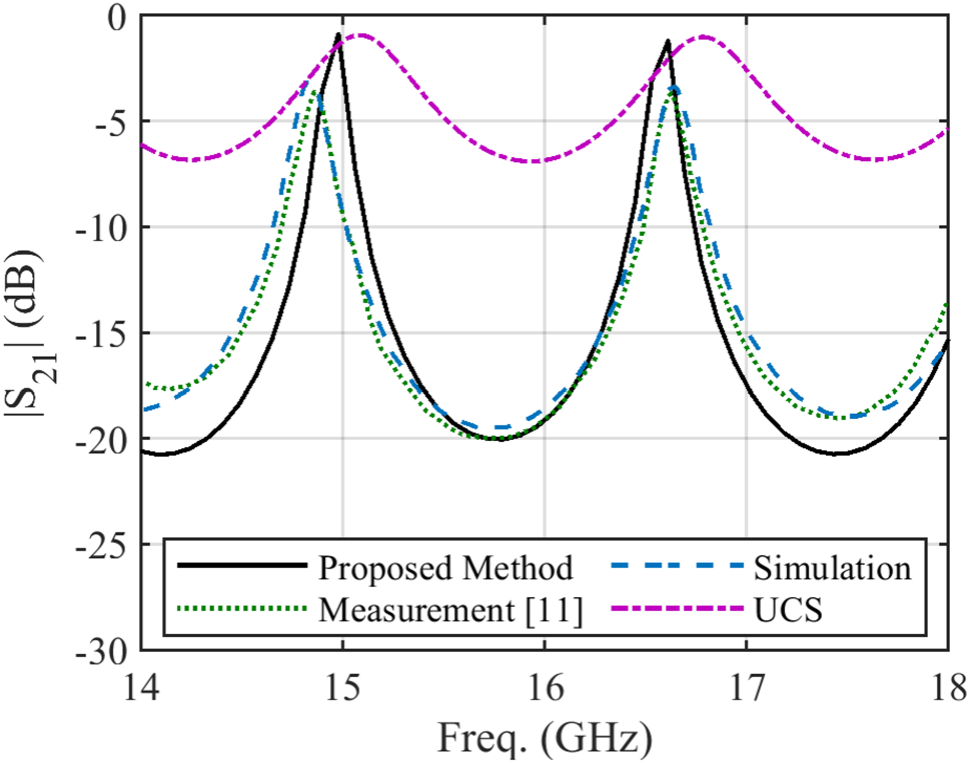
Magnitude of *S*_*21*_ of the linearly-tapered SIW.

By specifying the equivalent width, per-unit-length impedance and admittance are calculated using (17). The characteristic impedance of a SIW structure can be calculated using the closed-form formulas introduced in^26^. The calculated scattering parameters are shown in Figs. 10 and 11. As expected, the accuracy of the UCS method is not good since this method cannot be applied to a NUWG. A slight frequency shift, about 0.2 GHz, is seen between the results of the proposed method and measurement/simulation data. The peak magnitude deviation is about 4 dB for both *S*_*11*_ and *S*_*21*_ only at resonance frequencies. This is probably due to the transition between the SIW and SMA connectors and the fabrication imperfections, which are not addressed in the proposed method. However, the accuracy of the obtained results is acceptable over the wide frequency range of 14 GHz ≤ *f* ≤ 18 GHz.

Table 1 shows the required time *t*, number of segments *N* and frequency samples *FS* (to assess the efficiency), and condition number of the coefficient matrix *τ*. The condition number depends on the frequency. So, the worst case values are reported. It is helpful to note that the condition number is used to measure the sensitivity of the proposed method concerning the discretization error. More details about the acceptable range of the condition number can be found in^27,28^. For all examples, the condition number of the coefficient matrix is in an acceptable range, which means the low sensitivity of our proposed technique. The introduced method is implemented using a MATLAB-based program that runs on a PC with CPU core i5 @2.3 GHz & 4G RAM memory. The required running time of the proposed method is higher than the UCS technique. However, the accuracy of the introduced method is better than the UCS technique. Additionally, the proposed method shows robustness to different non-uniform guiding structures.

**Table 1:** Comparison of the important parameters of all studied examples.

		*t* (s)	FS	*τ*	*N*
Example 1	This work	0.06	50	6.8e6	100
	UCS	0.05	50	–	50
Example 2	This work	1.80	50	5.2e5	100
	UCS	0.80	50	–	50
Example 3	This work	2.8	100	1.2e5	100
	UCS	0.02	100	–	8
Example 4	This work	7.2	50	2.1e5	100
	UCS	1.60	50	–	100

## Conclusion

In this paper, the explicit Runge–Kutta technique is employed for analyzing the NUTLs and NUWGs. For this purpose, a system of equations is established based on TL modeling and its solution provides the magnitude and phase of the voltage and current propagating along the structure. Moreover, it is shown that the sensitivity of the proposed technique is suitable. Several theoretical and practical NUTLs and NUWG verify the proposed method's performance, and the results are compared with those obtained by other popular techniques such as the UCS approach. The method turns out to be simple in implementation. Examining the proposed method for a few structures shows that the introduced technique is accurate and stable.

## Data Availability

The datasets used and/or analyzed during the current study available from the corresponding author on reasonable request.
